# Does a three-degree hypoechogenicity grading improve ultrasound thyroid nodule risk stratification and affect the TI-RADS 4 category? A retrospective observational study

**DOI:** 10.20945/2359-3997000000608

**Published:** 2023-05-10

**Authors:** Ricardo Luiz Costantin Delfim, Lia Roque Assumpção, Flávia Paiva Proença Lobo Lopes, Patrícia de Fátima dos Santos Teixeira

**Affiliations:** 1 DASA Departamento de Radiologia Rio de Janeiro RJ Brasil DASA, Departamento de Radiologia, Rio de Janeiro, RJ, Brasil; 2 Universidade do Estado do Rio de Janeiro Departamento de Cirurgia Rio de Janeiro RJ Brasil Universidade do Estado do Rio de Janeiro, Departamento de Cirurgia, Rio de Janeiro, RJ, Brasil; 3 DASA Universidade Federal do Rio de Janeiro Departamento de Radiologia Rio de Janeiro RJ Brasil DASA, Universidade Federal do Rio de Janeiro, Departamento de Radiologia, Rio de Janeiro, RJ, Brasil; 4 Universidade Federal do Rio de Janeiro Faculdade de Medicina Programa de Pós--graduação em Endocrinologia Rio de Janeiro RJ Brasil Universidade Federal do Rio de Janeiro, Faculdade de Medicina, Programa de Pós--graduação em Endocrinologia, Rio de Janeiro, RJ, Brasil

**Keywords:** Thyroid nodule, thyroid neoplasms, ultrasonography, diagnosis, biopsy, cytology

## Abstract

**Objective:**

The aim of this study was to determine whether classifying hypoechogenicity in three degrees (mild, moderate, and marked) could improve the distinction between benign and malignant nodules and whether such an approach could influence Category 4 of the Thyroid Imaging Reporting and Data System (TI-RADS).

**Materials and methods:**

In total, 2,574 nodules submitted to fine needle aspiration, classified by the Bethesda System, were retrospectively assessed. Further, a subanalysis considering solid nodules without any additional suspicious findings (n = 565) was performed with the purpose of evaluating mainly TI-RADS 4 nodules.

**Results:**

Mild hypoechogenicity was significantly less related to malignancy (odds ratio [OR]: 1.409; CI: 1.086-1.829; p = 0.01), compared to moderate (OR: 4.775; CI: 3.700-6.163; p < 0.001) and marked hypoechogenicity (OR: 8.540; CI: 6.355-11.445; p < 0.001). In addition, mild hypoechogenicity (20.7%) and iso-hyperechogenicity (20.5%) presented a similar rate in the malignant sample. Regarding the subanalysis, no significant association was found between mildly hypoechoic solid nodules and cancer.

**Conclusion:**

Stratifying hypoechogenicity into three degrees influences the confidence in the assessment of the rate of malignancy, indicating that mild hypoechogenicity has a unique low-risk biological behavior that resembles iso-hyperechogenicity, but with minor malignant potential when compared to moderate and marked hypoechogenicity, with special influence on the TI-RADS 4 category.

## INTRODUCTION

Due to large-scale ultrasound tests performed worldwide, a high number of thyroid nodules are detected, especially nonpalpable ones ( 1 – 3 ). However, just 5%-15% of nodules are diagnosed as cancer ( 4 ). The ultrasound risk stratification system (RSS) has become the cornerstone for selecting nodules for fine needle aspiration (FNA) or follow-up. The nodule rate of malignancy (ROM) is determined by of the presence of suspicious signs, such as solidity, hypoechogenicity, calcifications, irregular margin, taller-than-wide shape, and extra-thyroid extension ( 4 – 9 ). One such feature, hypoechogenicity, is the focus of this study.

Hypoechogenicity, at any degree, is considered a high-sensitivity and low-specificity feature for malignancy ( 4 , 10 , 11 ). In contrast, marked hypoechogenicity has much higher specificity. Kim and cols. ( 12 ) described this finding as echogenicity lower than that of the previous strap muscle (ASM). However, discrepancies in grading hypoechogenicity patterns have been reported. Some authors have interpreted echogenicity related to the ASM or echogenicity similar to or lower than that of the ASM as marked hypoechogenicity ( 6 , 13 , 14 ). In contrast, Anderson and cols. ( 15 ) classified nodules as mildly, moderately, or very hypoechoic, but in relation to the thyroid parenchyma. Currently, most renowned systems ( 7 , 9 , 13 , 16 ) classify hypoechogenicity into two degrees, hypoechogenicity (related to parenchyma) and marked hypoechogenicity, as adopted by the American College of Radiology Thyroid Imaging Reporting and Data System (ACR TI-RADS) ( 9 ).

Given differences in hypoechogenicity patterns and their relationship to malignancy, our group ( 17 ) proposed three categories (mild, moderate, and marked) relative to the ASM. We showed that a higher association with malignant neoplasia exists when both moderately and markedly hypoechoic nodules are grouped together. More recently, Lee and cols. ( 18 ), showed that moderately and markedly hypoechoic nodules have a higher ROM than mildly hypoechoic ones.

Hypoechoic solid nodules, regardless of the hypoechogenicity degree, in the absence of additional suspicious features, are rated as ACR TI-RADS 4 (TR 4). Until now, no study has focused on the effect of grouping such nodules with different ROMs into a unique class.

Therefore, the aim of this study was to determine whether grading hypoechogenicity into three degrees might improve the distinction of benign and malignant nodules and whether such an approach could influence the TR4 category.

## MATERIALS AND METHODS

### Study design

A retrospective observational study was conducted with thyroid nodules submitted to FNA in Dasa's imaging centers. This study was approved by the ethics committee of the Clementino Fraga Filho University Hospital at the Federal University of Rio de Janeiro (approval number 053560/12), and it was done in accordance with the principles of the Declaration of Helsinki. In addition, informed written consent was obtained from all participants (Certificate of Presentation for Ethical Appreciation number 02266912.6.0000.5257).

The data were prospectively collected from 2574 solid and mixed nodules (≥5 mm) from 2,241 patients submitted to ultrasound-guided FNA for diagnostic purposes between January 2014 and December 2020. Completely cystic nodules were excluded. Only nodules with Bethesda ( 19 ) cytological categories 2 (benign), 5 (suspicious for malignancy), and 6 (malignant) were included. Suspicious for malignancy and malignant categories were described as “malignant” (n = 430). The final malignant diagnosis from this group was determined by postsurgical histology (n = 196) and the remaining ones by cytological assessment (n = 234). Benign samples were determined according to postsurgical histology (n = 86), benign cytology performed twice (n = 258), single benign cytology (n = 1307), and nodules in which no morphological changes over a 12-month follow-up were observed (n = 493).

### Ultrasonography and FNA

Thyroid ultrasounds and FNAs were carried out with 8-15 MHz multifrequency linear probes (Logiq S7 and S8 [GE] or Xsario SSA-660A and Applio 300, Toshiba, Minato, Japan), by a single radiologist, specialized in head and neck imaging and procedures (>25 years’ experience). All patients were referred for biopsy according to the criterion of each patient's physicians. The same scanning protocol was applied, regardless of the center. Ultrasound data recording was performed immediately after examination, with subsequent inclusion in the database. Data related to ultrasound patterns were reported before knowing the diagnosis.

For the execution of the FNA, a 20 mL plastic syringe coupled to a 30 x 7 mm (22 gauge) needle was used for aspiration. After introduction into the target lesion, zigzag and/or rotational movements were performed to obtain a sample. Usually, a single stint through the target lesion was enough.

### Cytological specimens

The specimens were smeared onto slides and fixed in 95% ethyl alcohol for Papanicolaou staining or fixed with 10% formaldehyde (cell block). All were subsequently submitted to cytopathological examination. Cytopathology was carried out by experienced cytopathologists with extensive expertise in thyroid diseases. The reports were categorized by the Bethesda system ( 19 ).

### Ultrasound analysis

**Figure 1:**
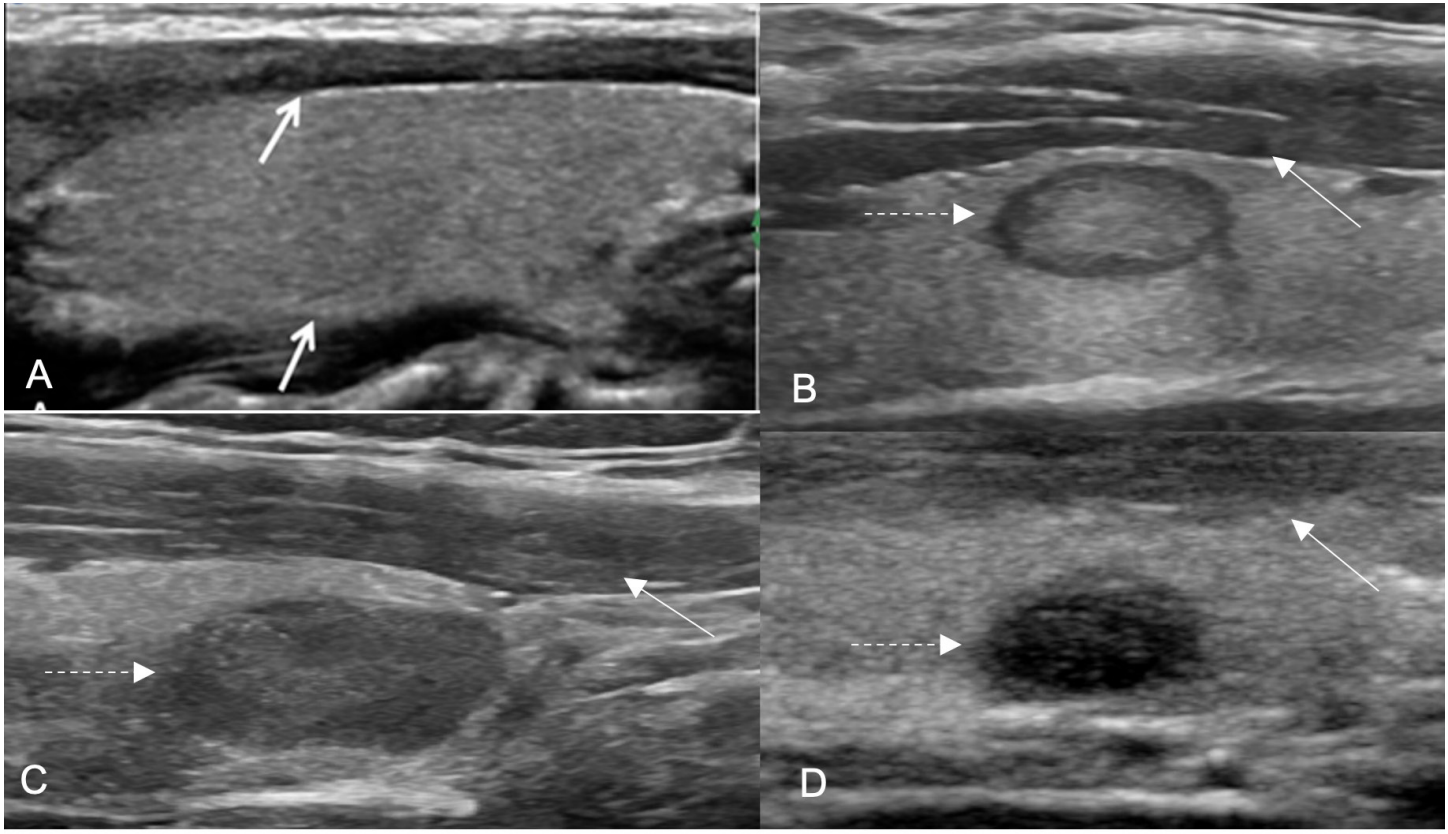
Hypoechoic nodules in different degrees. The nodule hypoechogenicity was compared to that of the anterior strap muscle (ASM) (white arrows). Longitudinal planes: ( **A** ) thyroid parenchymal echogenicity compared with that of the ASM; ( **B** ) mildly hypoechoic nodule; ( **C** ) moderately hypoechoic nodule and ( **D** ) markedly hypoechoic nodule (traced arrows).

The primary ultrasound criteria were composition and echogenicity ( 9 , 13 , 16 ). Regarding composition, every nodule was classified as solid (entirely solid or with cystic component ≤ 10%), predominantly solid (cystic component > 10% and < 50%), predominantly cystic (cystic component > 50%), cystic with solid mural area, and spongiform (multiple microcysts composing more than 50%). Echogenicity was classified as hyperechogenicity (nodule echogenicity > parenchyma) and isoechogenicity (nodule echogenicity = parenchyma). Both were set as iso-hyperechogenicity ( 9 ). Hypoechogenicity was determined as nodule echogenicity < parenchyma. The three respective degrees were graded as mild (nodule hypoechogenicity < ASM), moderate (nodule hypoechogenicity = ASM), and marked (nodule hypoechogenicity > ASM) ( 17 ). In nodules with heterogeneous textures, the predominant echogenicity pattern was considered the standard ( Figure 1 ).

Nodules with or without additional suspicious features, such as any sort of calcification, irregular margin, taller-than-wide shape, or extra-thyroid extension, were included. However, the thorough assessment of such features was not the scope of this study.

A subanalysis considering solid nodules without additional suspicious findings (n = 565) was performed to analyze the three-degree hypoechogenicity grading of the TR4 category further. ACR TI-RADS is a point-based RSS. Hypoechoic nodules (related to parenchyma) were assigned 2 points and markedly hypoechoic nodules were assigned 3 points.

### Interobserver agreement

One hundred cases were randomly selected for this calculation. A similar percentage (180/4,550 nodules [3.9%]) of cases was interpreted by other authors in another single observer article ( 20 ) because 3.9% of 2574 nodules is 100 nodules ( 20 ). In addition, 100 cases were previously analyzed for agreement interpretation using the ACR TI-RADS ( 21 ). Thereby, the echogenicity assessment, as designed in the Methods, was separately assigned through high-resolution ultrasound images by the primary observer (RLCD) and an external observer (RMP; >10 years in thyroid ultrasound). Both were blinded to outcomes.

### Statistical analysis

The continuous variables were expressed according to normal distribution as averages (±SD), if not, then they were expressed as median with 25 and 75 percentiles. Mann-Whitney U test and *t* test were used for these comparisons.

Categorical variables, as composition, echogenicity, and the three degrees of hypoechogenicity, were expressed in frequencies/percentages. The *x* ^2^ test was used to determine the difference between groups and to test the correlation with malignancy.

Logistic binary regression was performed including correlated independent variables, aiming to determine the strength of the relationship between the three-degree hypoechogenicity grading and malignancy. The odds ratio (OR) was calculated, in which the dependent variable was the malignant sample, with a 95% confidence interval (CI); the iso-hyperechoic nodules were the reference group.

The interrater reliability was set by Cohen's kappa index and Pearson's correlation test. Cohen's kappa index was interpreted as ≤ 0 = no agreement, 0-0.20 = slight agreement; 0.21-0.40 = fair; 0.41-0.60 = moderate; 0.61-0.80 = substantial; and 0.81-1.0 = almost perfect agreement ( 22 ). p < 0.05 was considered statistically significant.

Statistical analysis was done using of IBM SPSS (version 24.0).

## RESULTS

### Population and nodule data

Of 2,574 nodules, 83.3% were classified as benign and 16.7% as malignant. Among the benign, 90.3% were classified as nodular hyperplasia, 7.4% as Hashimoto's thyroiditis, 2.1% as nodular thyroiditis (Hashimoto's thyroiditis and nodular hyperplasia), and 0.2% as granulomatous thyroiditis. In the malignant group, among those diagnosed as suspicious for malignancy, 67% were suspicious for papillary carcinoma and 2.6% were suspicious for medullary carcinoma and lymphoma. Among those with malignant diagnosis, 28.8% were papillary carcinoma and 1.6% were medullary carcinoma, poorly differentiated, anaplastic or metastases.

The mean age of patients with cancer was significantly lower than that of those with benign nodules (46 *vs.* 49 years old, respectively, p < 0.001). The prevalence of women in both groups was high. Malignant nodules were significantly smaller than benign ones (1.20 *vs.* 1.65 cm in diameter, respectively; p < 0.001). Demographic data are shown in Table 1 .

**Table 1:** Distribution of nodules between malignant and benign samples, regarding age, sex, maximum dimension, composition, echogenicity, and three-degree hypoechogenicity grading

Demographics		Total % (n = 2,574)	Benign % (n) (n = 2,144)	Malignant % (n) (n = 430)	p value
Patients and nodules					
	Women	87.4 (2,250)	88.9 (1,906)	80.0 (344)	<0.001
Age, average (SD)		_	49.3 (13.2)	46.0 (15.0)	<0.001
Maximum dimension, median a		_	1.65 cm (1.20-2.30)	1.20 cm (0.90-1.80)	<0.001
US feature					
Composition					
	Solid	64.0 (1,648)	58.3 (1,250)	92.5 (398)	<0.001
	Predominantly solid	26.6 (685)	30.6 (657)	6.5 (28)	<0.001
	Predominantly cystic	2.6 (66)	3.1 (66)	0	<0.001
	Spongiform	4.8 (123)	5.7 (123)	0	<0.001
	Cyst with mural area	2.0 (52)	2.2 (48)	0.9 (4)	0.07
Echogenicity					
	Iso-hyperechogenicity	63.0 (1,621)	71.5 (1,533)	20.5 (88)	<0.001
	Hypoechogenicity	37.0 (953)	28.5 (611)	79.5 (342)	<0.001
Three-degree hypoechogenicity rating					
	Mild	44.5 (424)	54.8 (335)	26.0 (89)	0.01
	Moderate	32.9 (314)	29.8 (182)	38.6 (132)	<0.001
	Marked	22.6 (215)	15.4 (94)	35.4 (121)	<0.001

a 25-75 percentiles in parentheses (Mann-Whitney U test); SD: standard deviation (Student's t test). Percentages (X^2^ test).

Of the overall cohort, 64% (1,648/2,574) were solid nodules, of which 24% (398/1,648) were malignant. Within the mixed nodules group, 3.4% (32/926) were malignant. No significant difference between the malignant and benign groups (p = 0.07) was noted in cysts with solid mural area. Lastly, no cancer was present within predominantly cystic and spongiform nodules, despite echogenicity. Of cancers, 92.5% (398/430) were solid nodules. Table 1 exhibits the distribution of nodules according to composition.

### Echogenicity and three-degree hypoechogenicity grading and malignancy frequency

Regarding hypoechoic nodules (any degree), 35.9% (342/953) were malignant, in contrast with the 5.4% (88/1628) malignancy rate among iso-hyperechoic nodules (both p < 0.01).

As for the degree of hypoechogenicity, mildly hypoechoic nodules were the most frequent (44.5% [424/953]) in the sample. Moderately and markedly hypoechoic nodules were more prevalent in malignant nodules (both; p < 0.001) compared to mildly hypoechoic ones (26%; p = 0.01; Table 1 ).

### Three-degree hypoechogenicity grading within malignant tumor samples

**Figure 2:**
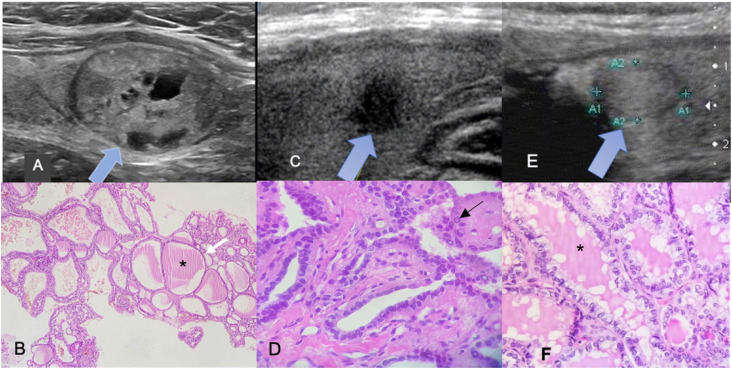
Ultrasound (US) and Histopathological specimens (hematoxylin-eosin stain) illustrations. US in longitudinal plans: ( **A** ) predominantly solid isoechoic hyperplastic nodule; TI-RADS 2. ( **B** ) Several macrofollicles lined by typical follicular cells, full of colloid substance*. ( **C** ) A classic subtype of papillary carcinoma represented as a markedly hypoechoic solid nodule, with irregular margin; TI-RADS 5. ( **D** ) A case of a papillary architecture that is lined by follicular cells that exhibit different nuclei sizes, irregular contours, nuclear enlargement and pseudoinclusions (arrow), and no colloid substance ( **E** ). A sample of a follicular subtype of papillary carcinoma is presented as an isoechoic solid nodule; TI-RADS 3. ( **F** ) An example of a follicular arrangement composed almost exclusively of follicles with atypical follicular cells that exhibits the same suspicious findings of classic subtype. In contrast with figure D, colloid substance* is noticed inside follicles.

Among the malignant nodules, 79.5% (342/430) were hypoechoic ( Table 1 ). Moderately and markedly hypoechoic nodules were significantly more prevalent within malignant samples, 30.7% (132/430) and 28.1% (121/430), respectively (both p < 0.001), when compared to mildly hypoechoic ones. A similar low malignancy frequency was found for both mildly hypoechoic nodules (20.7% [89/430]) and iso-hyperechoic nodules (20.5% [88/430]). Figure 2 exhibits nodules according to composition, echogenicity, and cytological specimens.

### Binary logistic regression analysis

**Figure 3:**
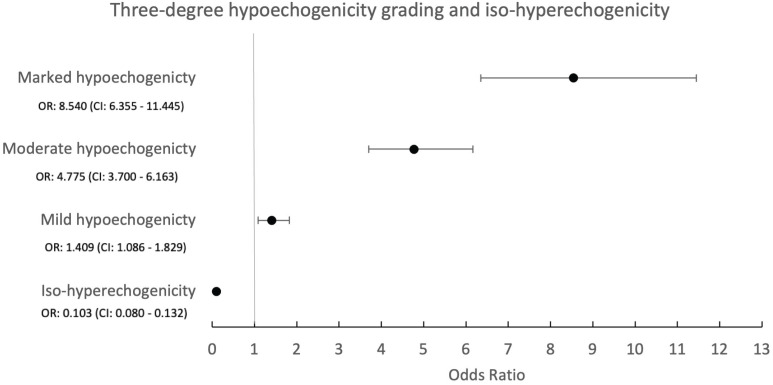
Forest plot for logistic regression according to three degrees of hypoechogenicity grading and iso-hyperechogenicity. The orange circles represent the odds ratio; the black lines represent the confidence interval (CI), with lower CI (left limit) and upper CI (right limit).

The mild hypoechogenicity grade had the lowest correlation with malignancy (OR: 1.409; CI: 1.086-1.829; p = 0.01), as compared to moderate (OR: 4.775; CI: 3.700-6.163; p < 0.001) and marked (OR: 8.540; CI: 6.355-11.445; p < 0.001) hypoechogenicity grades. Iso-hyperechogenicity was negatively related to malignancy (OR: 0.103; CI: 0.080-0.132; p < 0.001). Figure 3 exhibits these results.

### Subanalysis

Considering solid nodules without any additional suspicious finding (n = 565), ACR TI-RADS 3 (TR3) and TR4, only 6.9% (39/565) were malignant. Mild hypoechoic nodules were the most prevalent (55.4%) among hypoechoic nodules (TR4). In contrast, only 17.9% (7/39; p < 0.001) of iso-hyperechoic nodules (TR3) were malignant. For malignant samples, 82.1% (32/39) were hypoechoic, of which mild and moderate ones were the highest in prevalence, both 37.5% (12/32). No significant difference in malignancy was observed among mildly hypoechoic nodules, in contrast to the moderately (37.5% [12/32]; p < 0.001) and markedly hypoechoic ones (25% [8/32]; p < 0.001). Table 2 summarizes these results.

**Table 2:** Solid nodule (TR3/TR4) distribution regarding echogenicity and three-degree hypoechogenicity grading

Solid nodules (TR3 e TR4)		Total % (n = 565)	Benign % (n = 526)	Malignant % (n = 39)	p value
Echogenicity					
	Iso-hypoechogenicity	64.2 (363)	67.7 (356)	17.9 (7)	<0.001
	Hypoechogenicity	35.8 (202)	32.3 (170)	82.1 (32)	<0.001
Three-degree hypoechogenicity					
	Mild hypoechogenicity	55.4 (112)	58.8 (100)	37.5 (12)	<0.076
	Moderate hypoechogenicity	32.7 (66)	31.2 (54)	37.5 (12)	<0.001
	Marked hypoechogenicity	11.9 (24)	9.4 (16)	25.0 (8)	<0.001

*x* ^2^: square test.

**Figure 4:**
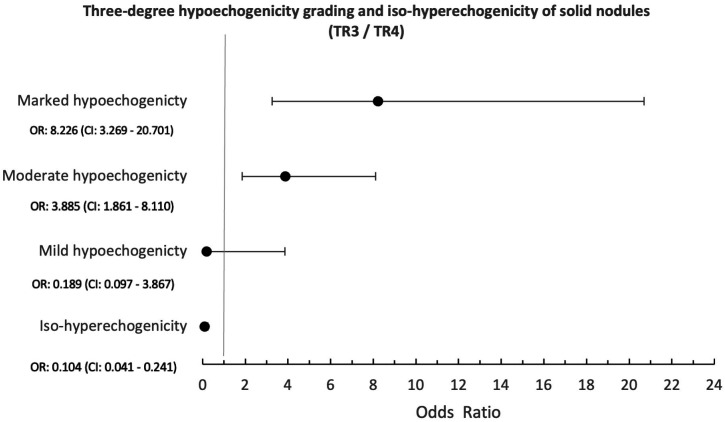
Forest plot sub-analysis. Logistic regression for the three degrees of hypoechogenicity grading and iso-hyperechogenicity grade of solid nodules (TR3 / TR4). The orange circles represent the odds ratio; the black lines represent the confidence interval (CI), with lower CI (left limit) and upper CI (right limit).

Based on binary logistic regression, for mildly hypoechoic nodules, no significantly association with malignancy was found (OR: 0.189; CI: 0.097-3.867; p = 0.08]), in contrast with moderately (OR: 3.885; CI: 1.861-8.110; p < 0.001]) and markedly hypoechoic nodules (OR: 8.266; CI: 3.269-20.701; p < 0.001]). Finally, iso-hyperechoic nodules were not positively associated with malignancy (OR: 0.104; CI: 0.041-0.241; p < 0.001]). Figure 4 shows these results.

### Interobserver reliability

The kappa index performed between the two reviewers was substantial to almost perfect (k = 0.80; CI: 0.73-0.85) for overall echogenicity. Among the subsets, the iso-hyperechoic (k = 0.95; CI: 0.75-1.15) and mildly hypoechoic nodules (k = 0.81; CI: 0.71-0.91) had almost perfect concordance. In contrast, the moderate hypoechoic nodules had the highest variance, with a moderate agreement (k = 0.66; CI: 0.56-0.77). In comparison, marked hypoechoic nodules were in moderate to substantial agreement (k = 0.80; CI: −0.70 to −0.90).

## DISCUSSION

In the current study, an increment in the ROM occurred as the nodules were progressively classified as mildly, moderately, and markedly hypoechoic, as found in the previous study by our group ( 17 ) and reported by Lee and cols. ( 18 ). Based on logistic analysis, the association between mild hypoechogenicity and malignancy was much lower than that of moderate and marked hypoechogenicity. This is in accordance with our previous series ( 17 ), where moderately and markedly hypoechoic nodules grouped together had a higher association with malignancy and were independently related to the likelihood of malignancy ( 17 ). Such data corroborate results published by Middleton and cols. ( 23 ), in which moderately and markedly hypoechoic nodules grouped together were reliable predictors of malignancy. Note that the risk of cancer development was also proportionally greater according to the degree of hypoechogenicity. These data show that three degree-hypoechogenicity grading can predict malignancy. In contrast, iso-hyperechogenicity was significantly more prevalent in benign nodules, and no association of iso-hyperechogenicity with malignancy was observed, as previously demonstrated ( 10 , 11 , 17 , 24 – 27 ). In our study, almost 90% of nodules submitted to FNA were benign, as already reported by other authors ( 17 , 26 – 28 ). Similar results (84%) were obtained by Lee and cols. ( 18 ). These data show the high number of unnecessary FNAs performed in clinical practice.

Among malignant tumors in our study, almost 80% were hypoechoic, as already reported by several authors ( 11 , 18 , 28 – 30 ). The moderate degree hypoechogenicity was the most prevalent among malignant nodules, around 30%, as previously observed by Lee and cols. ( 18 ). In that series, mildly hypoechoic nodules were most prevalent as well, but with a much lower ROM, compared to the moderately hypoechoic ones ( 18 ). Despite these findings, it is worth highlighting that both mild hypoechoic and iso-hyperechoic nodules had the almost malignancy prevalence in our series, even though iso-hyperechoic nodules have been associated with benign outcomes ( 10 , 17 , 24 , 25 , 31 , 32 ).

Significantly smaller cancers than those benign lesions were shown as well. We can infer that the higher detection rate of nonpalpable carcinomas was due notably to the widespread ultrasonography screening because this study sample comprised middle-income private patients with higher economic status ( 2 , 3 ).

Since 2011, the Korean guidelines ( 6 , 13 ) have ascribed nodules with hypoechogenicity similar to or lower than that of the ASM as markedly hypoechoic nodules. Similarly, Middleton and cols. ( 23 ) grouped moderately and markedly hypoechoic nodules together and assigned them 3 points in the ACR TI-RADS, unlike the ACR TI-RADS committee guidelines ( 9 ). The authors presumed that this combination could result in a reduction in the ROM of such markedly hypoechoic nodules, probably because of the lower malignant potential of moderately hypoechoic nodules. Supporting the classification of different degrees of hypoechogenicity and the ROM, Kwak and cols. ( 26 ) also reported a lower association with malignancy in hypoechoic nodules compared to markedly hypoechoic ones. Furthermore, the American Association of Clinical Endocrinologists guidelines ( 7 ) recommend that mildly hypoechoic nodules be included in the intermediate category together with iso-hyperechoic ones, which are allocated in the low-risk class by the American Thyroid Association ( 4 ). Nonetheless, in these studies, hypoechoic nodules include both mildly and moderately hypoechoic nodules according to our classification.

Such a relationship between degrees of hypoechogenicity and the ROM can be explained, because the tumor is composed of hypercellular tissues, which leads to cellular compaction and this, combined with the scarcity of colloid, causes lower sound reflection and, therefore, a hypoechoic appearance. Further, fibrosis can also enhance the degree of hypoechogenicity ( 27 , 29 – 31 ). This hypoechogenicity pattern is usually associated with the classical subtype of papillary carcinoma, whereas iso-hyperechoic appearance often occurs in the follicular subtype due to the exclusive or predominantly follicular component over the papillary component, which causes greater sound reflection ( 31 , 35 – 37 ). Thus, abundant colloid may explain the low-risk iso-hyperechogenicity aspect usually seen in hyperplasic nodules ( 23 , 32 , 35 ).

Considering the subanalysis in our study in which subgroups of TR3 and TR4 nodules were examined, the prevalence of malignancy among solid iso-hyperechoic nodules (TR3) was lower than 2%, less than the 3.9% found by Ha and cols. ( 30 ). In parallel, solid mildly hypoechoic nodules (TR4) were the most prevalent among hypoechoic nodules, even though not associated with malignancy, whereas moderately and markedly hypoechoic nodules were proportionally correlated with thyroid cancer. Concerning TR4 class, it is associated with an overall low risk. Middleton and cols. ( 23 ) found an aggregate risk of 9.1%, within the range initially attributed by the ACR TI-RADS committee (5% to 20%) ( 38 ). However, Di Fermo and cols. ( 39 ) obtained only 3.1% ROM. In contrast, the ROMs of the EU-TIRADS ( 16 ) and K-TIRADS ( 13 ) systems range between 6-17% and 15%-50%, respectively. In an attempt to minimize this broad ROM, some authors have proposed TI-RADS 4 subcategories ( 17 , 40 – 42 ). We have previously designated two subcategories in our RSS ( 17 ), TI-RADS 4A and TI-RADS 4B, and noted a difference in the ROM, corresponding to 7.8% and 35.3%, respectively. Additionally, others have separated TI-RADS 4 into three classes (A, B and C). Russ and cols. ( 41 ) had a 4% ROM in TI-RADS 4A and a negative association with malignancy. Barbosa and cols. ( 42 ) found a 28.1% ROM in ACR TI-RADS 4A a similar rate of the around the 22% from lower suspicion categories (ACR TI-RADS 2 and ACR TI-RADS 3), which was different from 55.6% from ACR TI-RADS 4B, in an indeterminate cytological sample.

The interobserver variability is a known factor that can directly influence ultrasound analysis of echogenicity. The agreement among authors is weak ( 31 , 43 – 47 ). Some technical factors, such as a poor technical approach by the operator, can generate relevant variations in the determination of echogenicity ( 31 , 43 ). Therefore, isoechoic nodules can be interpreted as mildly hypoechoic and vice versa, particularly in borderline images. These factors can generate significant variance in the determination of echogenicity. However, the overall echogenicity demonstrated a substantial to almost perfect concordance between our raters. In addition, iso-hyperechoic and mildly hypoechoic nodules had the highest reliability (almost perfect agreement), higher than measures shown by Lee and cols. ( 18 ). These authors also similarly demonstrated a higher concordance among mildly hypoechoic nodules when compared to the sternocleidomastoid muscle. However, the sternohyoid and sternum-thyroid muscles (ASM) were used as standard in our comparison, as was done by Kim and cols. ( 12 ), and afterwards were adopted by others RSSs ( 7 , 9 , 13 , 16 , 26 , 27 ). Further studies may support whether a relevant difference exists between these approaches.

To our knowledge, our group was the first to evaluate the role of three-degree hypoechogenicity grading, comparing it to that of the ASM, in thyroid nodule management ( 17 ). Additionally, comparing nodule echogenicity to that of the ASM may be advantageous in evaluations performed in the context of lymphocytic thyroiditis (Hashimoto's thyroiditis), a highly prevalent disorder in which the parenchyma is usually hypoechoic and heterogeneous, which makes it challenging to analyze ( 15 , 31 ). Hence, this technique is practical and reproducible, and it has no additional costs and can provide greater diagnostic accuracy by conventional ultrasound in predicting malignancy. However, further studies are needed to confirm this statement.

This study has several limitations. Although cytology is not considered the best standard for the outcome as compared with histology, it was adopted for two reasons: One of the objectives was to determine whether mild hypoechogenicity was poorly correlated with thyroid cancer, as proven. Therefore, most nodules were not expected to be submitted to surgery, which would considerably reduce our sample if histology were used as the criterion standard. Even though misclassification is possible, a high agreement (98.5%) between the Bethesda classification and histopathology was observed in our previous study ( 17 ), corroborating this strategy. Regarding the exclusive selection of Bethesda category 2, 5, and 6 nodules, we wished to minimize the influence of the lack of diagnostic assessment from the nondiagnostic and low accuracy of undetermined categories because heterogeneity in the ROM of indeterminate categories is an important factor, in both the ultrasound and cytological aspects ( 48 – 50 ). Finally, the analysis was done exclusively by one operator, who did not know the outcome. Despite this, an interrater agreement between two reviewers was estimated to strengthen our results. Furthermore, the intraobserver variation was minimized by applying the same exam and procedure protocols and collecting data.

In synthesis, conventional ultrasonography is a noninvasive and optimal cost-benefit technique for stratifying thyroid nodules. Albeit most thyroid nodules undergo FNA, the majority have a benign diagnosis. Consequently, improvement on risk stratification is needed. The key role of a three-degree hypoechogenicity grading system was established according to our results. Mildly hypoechoic nodules were the most prevalent among hypoechoic nodules, although less associated with malignancy. No association was found between mildly hypoechoic solid nodules and cancer. Moreover, mildly and moderately hypoechoic nodules are rated together in the TR4 category ( 9 ), albeit moderately hypoechoic nodules were the most common among malignant tumors in our study. Therefore, mildly hypoechoic solid nodules should be allocated in a lower risk category (TR4-A) to avoid unnecessary FNA procedures, although further studies are needed to confirm this recommendation.

In conclusion, stratifying hypoechogenicity into three degrees influences the confidence in the assessment of the rate of malignancy in thyroid nodules, indicating that mild hypoechogenicity has a unique low-risk biological behavior that resembles iso-hyperechogenicity but with minor malignant potential when compared to moderate and marked hypoechogenicity, with special influence on TI-RADS 4 category.
